# Complete Genome Sequence of Erwinia amylovora Strain 99east-3-1, Isolated from Pyrus sinkiangensis in China

**DOI:** 10.1128/mra.00161-23

**Published:** 2023-06-14

**Authors:** Nuoya Fei, Yuwen Yang, Bo Song, Xiaofeng Zhu, Wei Guan, Tingchang Zhao

**Affiliations:** a State Key Laboratory for Biology of Plant Diseases and Insect Pests, Institute of Plant Protection, Chinese Academy of Agricultural Sciences, Beijing, China; b College of Life Sciences, Jilin Normal University, Siping, China; c Institute of Plant Protection, Xinjiang Academy of Agricultural Sciences/Key Laboratory of Integrated Pest Management on Crops in Northwestern Oasis, Ministry of Agriculture, Urumqi, China; University of Arizona

## Abstract

Here, we report the complete genome sequence of Erwinia amylovora strain 99east-3-1, which was isolated from Pyrus sinkiangensis in Xinjiang Uygur Autonomous Region, China.

## ANNOUNCEMENT

Fire blight caused by Erwinia amylovora is a destructive bacterial disease that seriously endangers the production of rosaceous plants (1). However, genome resources for E. amylovora from China are rare. Here, we report a complete genome sequence of E. amylovora (strain 99east-3-1) from China. A sample of fire blight on Pyrus sinkiangensis was collected on 16 July 2021 in Bayingolin Mongolian Autonomous Prefecture (Xinjiang Uygur Autonomous Region; 41°40′24″N, 86°5′30″E). The infected branch tissue was cut, sterilized, and ground. After static precipitation, the supernatant was streaked on a nutrient agar (NA) plate and incubated at 28°C for 36 h. Single colonies were selected and identified by sequences of 16S rRNA gene fragments (the primers 27F/1492R were used; see “Data availability”). Strain 99east-3-1 was identified as E. amylovora and chosen for DNA extraction.

A single colony was grown in nutrient broth for 24 h, and 15 mL of the bacterial suspension was centrifuged. Genomic DNA was extracted using SDS method (2). The DNA was submitted to Tsingke Biotechnology (Beijing, China) for sequencing, after testing using the NanoDrop One spectrophotometer (Wilmington, DE), Qubit 3.0 fluorimeter (Life Technologies, Carlsbad, CA, USA), and gel electrophoresis. For Nanopore sequencing, DNA libraries were prepared utilizing a single library preparation method with the SQK-LSK109, EXP-NBD104, and 114 kits (Oxford Nanopore Technologies, Oxford, UK). The Qubit 2.0 fluorimeter was used for DNA quantification and library dilution. The library was sequenced using the PromethION sequencer (R9.4 chip; Oxford Nanopore Technologies). Library insert fragments were detected using the Agilent 2100 Bioanalyzer system. The Nanopore long reads were subjected to quality control using NanoPlot v1.15.0 with a threshold quality (Q) value of >7 (3), resulting in 54,987 high-quality reads with an average read length of 18,186.4 bp and an *N*_50_ value of 20,514 bp. For Illumina sequencing, DNA libraries were prepared utilizing a single library preparation method based on the Illumina Nextera kit and sequenced on the NovaSeq 6000 platform (4–9). We obtained paired-end reads (2 × 150 bp), totaling 8,114,076 reads covering a total of 1.22 Gb clean data (Q20, 97.25; Q30, 92.27). Unicycler v0.4.9 (10) was used to assemble the clean reads. Pilon v1.23 (11) was used to correct the assembled genome, using the second-generation data to obtain a final genome with higher accuracy. The average sequencing depth of Illumina sequencing was 308.54×, and the average sequencing depth of Nanopore sequencing was approximately 257.82×.

The complete genome of 99east-3-1 contains a circular chromosome of 3,799,623 bp with a GC content of 53.62% and a plasmid of 28,283 bp with a GC content of 50.21%. The whole-genome sequence (WGS) was annotated using the NCBI Prokaryotic Genome Annotation Pipeline (12) (Table 1). The genome of 99east-3-1 has a high sequence identity with the genome of representative E. amylovora strains ATCC 49946 (GenBank accession number NC_013971.1) and CFBP1430 (NC_013961.1), with an average nucleotide identity (ANI) of 99.98% (13). The ANI genome comparisons were performed using EzBioCloud (https://www.ezbiocloud.net/tools/ani). Default parameters were used for all software.

**TABLE 1:** Genome annotation statistics of 99east-3-1

Characteristic	No.^*a*^
Genes	3,478
CDSs^*b*^	3,361
rRNAs (5S, 16S, 23S)	8, 7, 7
Complete rRNAs (5S, 16S, 23S)	8, 7, 7
tRNAs	76
Pseudogenes	88
CRISPR arrays	2

a Determined using PGAP.

b CDSs, coding DNA sequences.

### Data availability.

The whole-genome sequencing project of 99east-3-1 has been deposited at NCBI GenBank under the accession number NZ_CP117554.1, BioProject accession number PRJNA893880, and BioSample accession number SAMN31435602. The 16S rRNA gene fragments have been deposited under the GenBank accession number OQ851893. The raw sequences have been deposited in the SRA under the accession number SRR22031458.
